# Two decades of aortic coarctation treatment in children; evaluating techniques

**DOI:** 10.1007/s12471-020-01513-y

**Published:** 2020-11-11

**Authors:** E. J. Dijkema, L. Dik, J. M. P. Breur, G. T. Sieswerda, F. Haas, M. G. Slieker, P. H. Schoof

**Affiliations:** 1 Department of Pediatric Cardiology, Wilhelmina Children’s Hospital (WKZ), University Medical Center Utrecht, Utrecht, The Netherlands; 2 Department of Cardiology, University Medical Center Utrecht, Utrecht, The Netherlands; 3 Department of Cardiothoracic Surgery, Wilhelmina Children’s Hospital (WKZ), University Medical Center Utrecht, Utrecht, The Netherlands

**Keywords:** Congenital heart disease, Coarctation, Cardiothoracic surgery, Endovascular stent placement

## Abstract

**Objective:**

This study focuses on the evolution of treatment techniques for aortic coarctation in children and assesses long-term morbidity.

**Methods:**

This retrospective cohort study evaluates patients treated for native aortic coarctation, with at least 7 years of follow-up. To assess time-related changes, three time periods were distinguished according to year of primary intervention (era 1, 2 and 3). Operative and long-term follow-up data were collected by patient record reviews.

**Results:**

The study population consisted of 206 patients (177 surgical and 29 catheter-based interventions), with a median follow-up of 151 months. Anterior approach with simultaneous repair of aortic arch and associated cardiac lesions was more common in the most recent era. Median age at intervention did not change over time. Reintervention was necessary in one third of the cohort with an event-free survival of 74% at 5‑year and 68% at 10-year follow-up. Reintervention rates were significantly higher after catheter-based interventions compared with surgical interventions (hazard ratio [HR] 1.8, 95% confidence interval [CI] 1.04–3.00, *p* = 0.04) and in patients treated before 3 months of age (HR 2.1, 95% CI 1.27–3.55, *p* = 0.003). Hypertension was present in one out of five patients.

**Conclusion:**

Nowadays, complex patients with associated cardiac defects and arch hypoplasia are being treated surgically on bypass, whereas catheter-based intervention is introduced for non-complex patients. Reintervention is common and more frequent after catheter-based intervention and in surgery under 3 months of age. One fifth of the 206 patients remained hypertensive.

## Introduction

Coarctation of the aorta (CoA) is a relatively common congenital anomaly, responsible for 5–10% of all congenital heart defects [1–3]. The first documented therapy for CoA was in 1945; surgical resection of the narrowed segment followed by end-to-end anastomosis [4]. Over the past decades surgical techniques have been modified and catheter-based intervention of CoA has been introduced as an alternative to surgery in both children and older patients [2, 5, 6]. Despite anatomically successful repair, patients are reported to have a reduced life expectancy, attributed to complications later in life, including hypertension, aneurysm formation, aortic dissection and rupture, and reinterventions [1, 3, 6, 7]. Therefore, long-term follow-up is recommended. This study focused on the long-term evolution of techniques in patients treated for native CoA. We present our experience from 1986 to 2010.

## Methods

### Study population

Patient characteristics are depicted in Tab. 1. We included patients treated at our centre (the Wilhelmina Children’s Hospital, Utrecht, the Netherlands) for native CoA at the age of <18 years with at least 7 years of follow-up (= intervention before 2010). Patients were grouped based on year of treatment: era 1 (1987–1994), era 2 (1995–2002) and era 3 (2003–2010), and split according to treatment strategy: surgery (end-to-end anastomosis, extended end-to-end anastomosis, patch angioplasty, aortic arch reconstruction and interposition graft) versus catheter-based interventions (balloon angioplasty with or without endovascular stent placement). Data on long-term follow-up were acquired from patients’ medical records. A hypoplastic aortic arch was defined as a proximal or distal transverse arch diameter less than 60% or 50% of the diameter of the ascending aorta, respectively [8].

**Table 1:** Baseline characteristics of the study population

		Overall	Group 1	Group 2	Group 3	*p*-value
			(1987–1994)	(1995–2002)	(2003–2010)	
		*n* = 206	*n* = 59	*n* = 58	*n* = 89	
Age at intervention (days)		30 (2–6413)	35 (2–5579)	28 (4–5231)	26 (5–6413)	0.54
	Infants	159 (77%)	48 (81%)	46 (79%)	65 (73%)	0.45
	Young children	36 (17%)	10 (17%)	9 (16%)	17 (19%)	0.85
	Adolescents	11 (5%)	1 (2%)	3 (5%)	7 (8%)	0.26
Intervention <3 months of age		135 (66%)	40 (68%)	38 (66%)	57 (64%)	0.90
Age at study (years)		15.5 (7.0–35.5)	22.7 (7.0–30.8)	17.4 (7.0–35.5)	11.0 (7.0–25.3)	<0.01
Sex (male)		137 (67%)	36 (61%)	42 (72%)	57 (64%)	0.93
Follow-up (months)		152 (75–347)	268 (83–347)	205 (79–274)	117 (75–166)	<0.01
Associated lesions		147 (71%)	42 (71%)	38 (65%)	67(75%)	0.38
	Bicuspid aortic valve	80 (39%)	22 (37%)	22 (38%)	36 (40%)	0.92
	Hypoplastic aortic arch	43 (21%)	6 (10%)	8 (14%)	29 (33%)	<0.01
	Ventricular septal defect	78 (38%)	24 (41%)	19 (33%)	35 (39%)	0.63
	Patent ductus arteriosus	44 (21%)	16 (27%)	3 (5%)	25 (28%)	<0.01
Haemodynamically significant comorbidity^a^		114 (55%)	31 (53%)	23 (40%)	59 (66%)	<0.01

Data are presented as median (range) or number of patients (percentage)

Group 1, treatment between 1987 and 1995. Group 2, treatment between 1996 and 2002. Group 3, treatment between 2003 and 2010

^a^Haemodynamically significant comorbidity requiring repair

The Medical Ethics Committee of the University Medical Center Utrecht has decided that the official approval of this study by the Medical Ethics Committee was not required.

### Treatment strategies

Surgical repair with end-to-end anastomosis was performed through either a lateral thoracotomy without use of extracorporeal circulation or a median sternotomy with the use of extracorporeal circulation, in the presence of hypoplastic aortic arch and/or associated cardiac defects. In the early years, patch angioplasty was performed through a lateral thoracotomy without the use of extracorporeal circulation. Over the years, a primary approach was favoured with a median sternotomy and aortic arch reconstruction with a patch and use of extracorporeal circulation, defined as arch reconstruction. Catheter-based treatment consisted of balloon angioplasty with or without stent placement. The technique was introduced at our centre in 1990 and supplemented with stent placement in 2008.

### Follow-up data

Follow-up focused on reintervention rate (catheter-based or surgical), presence of hypertension and aortic arch aneurysm formation in the patients who underwent computed tomography (CT) or magnetic resonance imaging during follow-up. Reference values for hypertension were based on those reported by Wuhl et al. [9].

### Statistical analysis

Statistical analyses were performed using IBM SPSS Statistics, version 25. Dichotomous variables were compared using Pearson’s chi-squared test. For every continuous variable, the Kolmogorov-Smirnov test was used to determine whether the values were normally distributed. Normally distributed continuous variables were compared using ANOVA and the mean value was described in the results. If the variable was not normally distributed, the Kruskal-Wallis test was used and the median value described in the results. Reintervention-free survival analysis was performed using a Kaplan-Meier curve. We used a log-rank test to assess differences in reintervention-free survival. Test results with a *p-*value <0.05 were considered significant.

## Results

### Study population

**Fig. 1:**
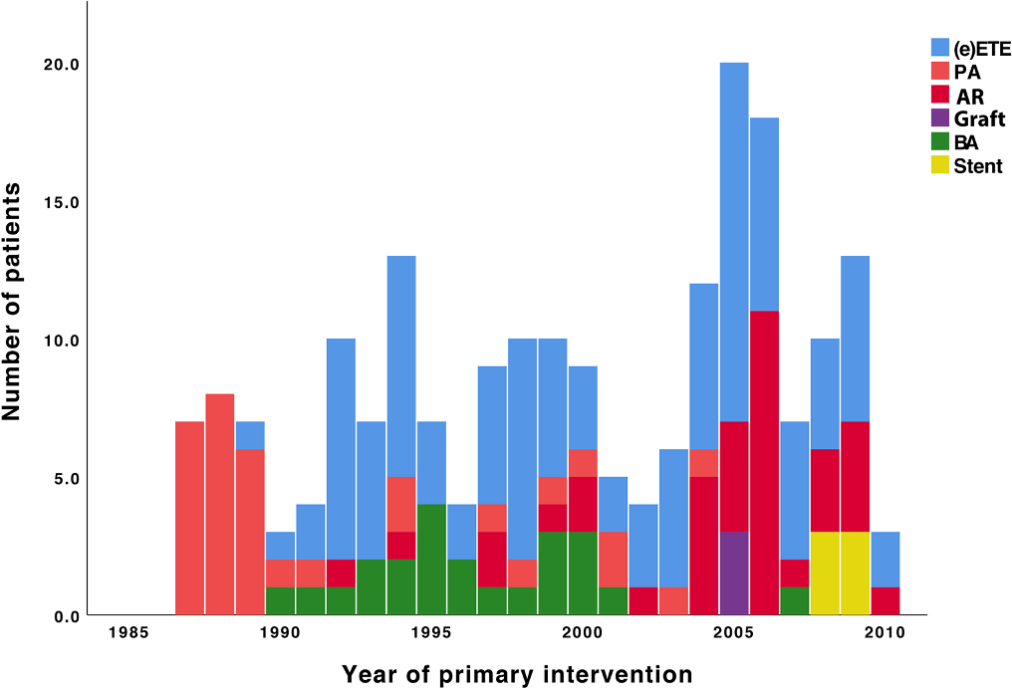
Various treatment strategies over the years. *(e)ETE* (extended) end-to-end anastomosis, *PA* patch angioplasty, *AR* aortic arch reconstruction, *BA* balloon angioplasty

A total of 206 patients (67% male) had a follow-up of at least 7 years. Patient characteristics are summarised in Tab. 1. Associated cardiac defects were reported in 147 patients (ventricular septal defect [38%], bicuspid aortic valve [39%], patent ductus arteriosus [21%] and hypoplastic aortic arch [21%]) and 114 (55%) patients had haemodynamically significant comorbidities. Two thirds of patients were treated in the first three months of life. Median age at intervention was 30 days (range 2–6413). Surgical treatment was performed in 177 patients (75 with end-to-end anastomosis, 29 with extended end-to-end anastomosis, 33 with patch angioplasty through lateral thoracotomy without use of extracorporeal circulation, 37 with aortic arch reconstruction through median sternotomy with use of extracorporeal circulation, hypothermia and selective antegrade cerebral perfusion and 3 with interposition graft) and catheter-based intervention in 29 patients (6 with and 23 without stent) (Fig. 1; Tab. 2). Median age at follow-up was 15.5 years (range 7–35). Interval between initial intervention and last follow-up was 151 (range 75–347) months. CT or magnetic resonance imaging was performed in 42% of patients after a minimum follow-up of 7 years. All of the 127 patients (11 adults and 116 children) who did not have CT or magnetic resonance imaging underwent echocardiographic imaging of the aorta to assess aneurysm formation.

**Table 2:** Treatment strategies used for primary treatment

	Overall	Group 1	Group 2	Group 3	*p*-value
		(1987–1994)	(1995–2002)	(2003–2010)	
	*n* = 206	*n* = 59	*n* = 58	*n* = 89	
End-to-end anastomosis	104 (50%)	25 (42%)	31 (54%)	48 (54%)	0.34
Patch angioplasty	33 (16%)	25 (42%)	6 (10%)	2 (2%)	<0.001
Aortic arch reconstruction	37 (18%)	2 (4%)	6 (10%)	29 (33%)	<0.001
Interposition graft	3 (1%)	–	–	3 (3%)	NA
Balloon angioplasty alone	23 (11%)	7 (12%)	15 (26%)	1 (1%)	<0.001
Balloon + stent placement	6 (3%)	–	–	6 (7%)	NA

*NA* not available

### Treatment

Data on CoA treatment in the three different treatment eras are presented in Tab. 2. The number of patients increased significantly over the years. Recently, more patients were treated for hypoplastic arch (*p* = 0.001) and had associated cardiac defects (*p* = 0.006). Mean age at initial intervention decreased from 30 to 26 days (*p* = 0.54) and overall two thirds of patients were treated in the first three months of life. Tab. 3 provides data on treatment strategies (surgery versus endovascular treatment) over the different age groups at time of treatment.

**Table 3:** Treatment strategies used in different age groups at time of treatment

	Overall	Surgery	Endovascular
	*n* = 206	*n* = 177	*n* = 29
Infant (0–1 yr)	159	150 (94%)	9 (6%)
Young (1–10 yr)	36	22 (61%)	14 (39%)
Adolescent (10–18 yr)	11	5 (45%)	6 (55%)

### Follow-up

Data on long-term follow-up is presented in Tab. 4.

**Table 4:** Long-term outcome after treatment of native coarctation of the aorta

		Overall	Group 1	Group 2	Group 3	*P*-value
		(*n* = 206)	(*n* = 59)	(*n* = 58)	(*n* = 89)	
Reintervention		83 (40%)	21 (36%)	31 (53%)	32 (36%)	0.07
Hypertension		42 (20%)	14 (24%)	14 (24%)	14 (16%)	0.35
Aneurysm formation						
	CT or MR imaging	86 (42%)	35 (59%)	30 (52%)	21 (24%)	<0.01
	Aneurysm found	7 (8%)	2 (6%)	3 (10%)	2 (10%)	0.80

Group 1, treatment between 1987 and 1995. Group 2, treatment between 1996 and 2002. Group 3, treatment between 2003 and 2010

*CT* computed tomography, *MR* magnetic resonance

#### Reintervention

Reinterventions were reported in 83 (40%) patients: residual or recoarctation in 78 and aortic aneurysm in 5 patients. The interval between initial treatment and first reintervention ranged from 0.2 to 322 months. Event-free survival was 74% at 5‑year and 68% at 10-year follow-up, irrespective of treatment era (*p* = 0.11 and *p* = 0.16 respectively). Patients who had a catheter-based intervention (with or without stent placement) had a significantly higher risk of reintervention compared with surgically treated patients (hazard ratio [HR] 1.8, 95% confidence interval [CI] 1.04–3.00, *p* = 0.04).

**Fig. 2:**
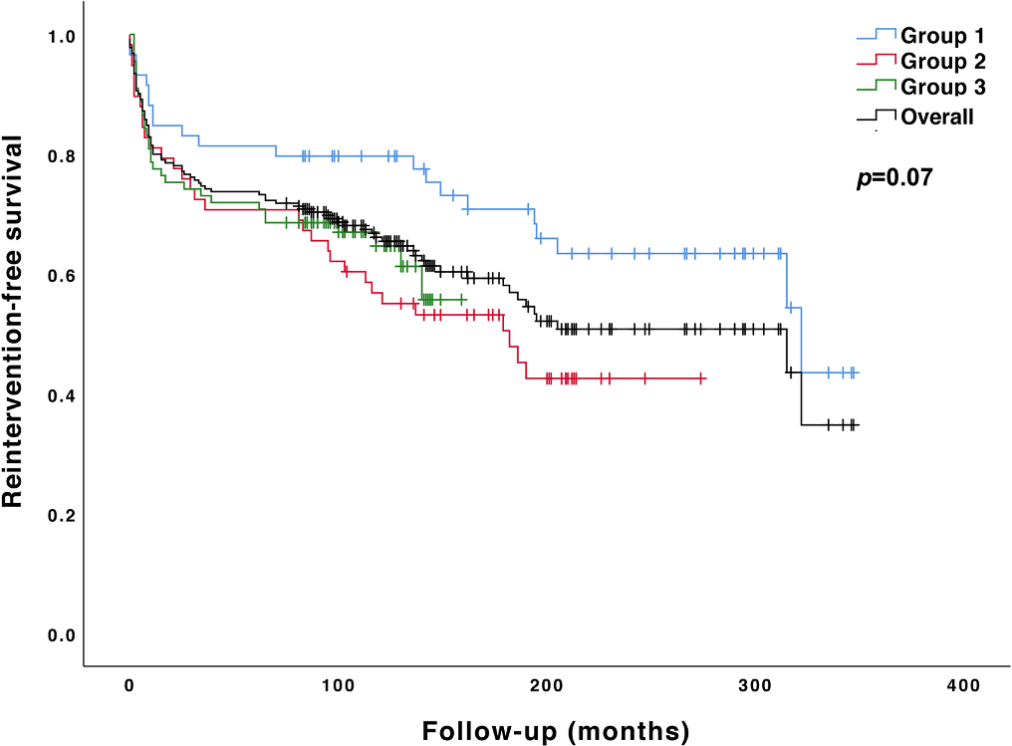
Kaplan-Meier curve showing reintervention-free survival in the different treatment periods. *Blue line*: Group 1 (1987 and 1995). *Red line*: Group 2 (1996 and 2002). *Green line*: Group 3 (2003 and 2010). *Black line*: mean reintervention-free survival (*p* = 0.07)

**Fig. 3:**
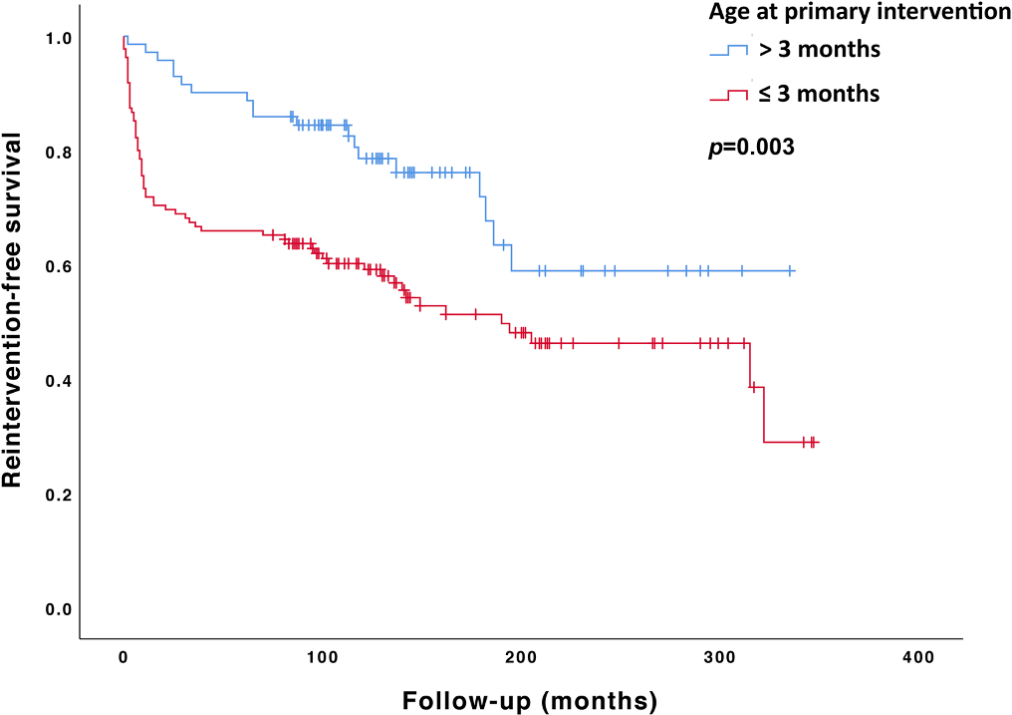
Kaplan-Meier curve of the reintervention-free survival in patients comparing treatment <3 (*blue line*) and >3 months of age (*red line*) (*p* < 0.01)

At 10-year follow-up, event-free survival was 71% for surgically treated patients compared with 50% for patients with catheter-based interventions (*p* = 0.03). Differences in reintervention rates across the different treatment periods were not statistically significant (*p* = 0.07) (Fig. 2). Patients treated before 3 months of age had a significantly higher risk of reintervention compared with patients treated later in life (HR 2.1, 95% CI 1.27–3.55, *p* = 0.003) (Fig. 3).

#### Hypertension

Hypertension was reported in 42 (20%) patients with equal prevalence across the three treatment periods (*p* = 0.35). It occurred often in catheter-based interventions compared to surgical interventions (38% versus 18%, respectively, *p* = 0.01).

#### Aneurysms

In 86 patients in whom the aortic arch was studied with CT or magnetic resonance imaging, aneurysm formation was described in 7 (8%) patients, of which 5 had aneurysm formation after primary treatment (1 patch angioplasty, 2 end-to-end anastomosis and 2 catheter-based) and 2 after reintervention (both catheter-based). Five aneurysms required reintervention (2 surgical and 3 catheter-based). No differences in prevalence of aneurysm formation were found between the different treatment periods (*p* = 0.80).

## Discussion

### Treatment

We found that over time the number of patients had increased. The cause of this increase is possibly attributed to changes in patient referral and improved diagnostics [10–12].

In contrast to patients treated in the early era in whom resection and end-to-end anastomosis was the favoured approach, recently performed operations were done through a median sternotomy with the use of extracorporeal circulation, hypothermia and selective antegrade cerebral perfusion, and confined to patients with arch hypoplasia and associated cardiac lesions.

In the past, the aortic arch was expected to show catch-up growth after single end-to-end anastomosis [13]. However, this did not appear to happen consistently [14]. Moreover, whether the aortic arch grows with the patient after CoA repair might be related to the extent of arch hypoplasia [15]. Several studies have assessed growth of the aortic arch after isolated end-to-end repair of CoA in association with arch hypoplasia and found that normal blood flow over the former coarctation site does not always guarantee normal aortic arch growth afterwards [14, 15]. Therefore, the threshold to concomitantly address the arch has become lower. This more radical approach is reflected in our study group.

Balloon angioplasty was introduced as an alternative therapy to surgery in the 1980s [16]. Thought to be a saver and less invasive treatment modality, balloon angioplasty was widely used for repair of CoA. However, after long-term follow-up, several studies showed that balloon angioplasty was associated with a higher incidence of aneurysm formation and recoarctation causing it to fall from grace as treatment for native CoA [15, 17, 18]. More recently, endovascular stent placement has been introduced. Stent placement is thought to have the benefits of balloon angioplasty (i.e. a less invasive treatment), but lower rates of recoarctation due to the stiff stent material placed at the coarctation site [19]. However, stent implantation in young children remains controversial, as it requires frequent redilatation to accommodate the growing aorta. Furthermore, stent placement in young children is associated with a high incidence of intimal proliferation and restenosis and a risk of post-stent aneurysm formation [20]. Therefore, stent placement is reserved as a treatment for older children with no concomitant cardiac morbidity, as is reflected in our study group. Despite improved diagnosis, age at referral did not change over time and was similar in all 3 eras.

### Follow-up

Residual lesions were a frequent cause of reintervention with a 68% event-free survival at 10-year follow-up. In catheter-based (both balloon angioplasty and stent placement) treated patients, the rate of reintervention was higher. Explanatory, pioneer experience with exclusive use of balloon dilatation without the use of stent may have caused this high rate of reinterventions [15, 17, 21]. Supplemental stenting was only introduced in 2008. Apparently, dilatation of the coarctation is less effective than surgical repair, as reinterventions after surgery were less common. Modification of surgical technique from lateral approach without extracorporeal circulation to anterior approach with extracorporeal circulation did not change reintervention rates over time. Our reintervention rates of 40% are comparable with other studies [1, 2, 4, 7, 19]. However, in the available literature reintervention rates vary widely.

Brown et al. described reintervention rates of 7.2, 14.3 and 23.4% at 10, 20 and 30 years after initial surgical treatment in a cohort of 819 patients treated at a mean age of 17 years [22]. In contrast, Choudhary et al. showed a reintervention rate of 31% in patients treated surgically at a mean age of 5 years, with a follow-up of 26 years [7]. Our relatively higher reintervention rates might be explained by a younger age at intervention in our cohort. Repair before the age of 1 year has been identified as a risk factor for recoarctation and reintervention [6, 22]. Indeed, our study confirms a significant risk of reintervention for patients treated before 3 months of age.

Hypertension occurred more often in patients following catheter-based intervention compared with surgery. Comparative data on hypertension in long-term follow-up is very limited. Chiu et al. described equal prevalence of hypertension in surgical and catheter-based interventions at 10-year follow-up. However, the lack of baseline patient characteristics prevented further comparison of the results [23]. Older age at time of intervention has been identified as a risk factor for hypertension in long-term follow-up [24]. Given that a large number of the patients with a catheter-based intervention were treated at older age, this might explain the observed difference in prevalence of hypertension.

We observed a low rate of CT and magnetic resonance imaging across the study population, especially in the most recent era, which may have resulted in a relatively low rate of aneurysm formation in our study group. The lack of CT and magnetic resonance imaging might be explained by the fact that these patients have not yet reached adulthood, resulting in a large number of patients who only received echocardiographic imaging during follow-up. Nevertheless, the most recent guidelines suggest that magnetic resonance (or if necessary CT) imaging should be performed at least every 5 years as soon as the patients is old enough to undergo scanning without the need for sedation [11]. Furthermore, in a smaller substudy on long-term outcome (>10 years) after coarctation repair, we found that many patients after coarctation treatment had been lost to follow-up [25]. In several of these patients, magnetic resonance imaging revealed recoarctation or aneurysm formation. Our current data show that the need for reintervention persisted up to 26 years after initial treatment, underscoring the importance of continued follow-up.

### Limitations

Comparison of the different treatment periods might be influenced by differences in length of follow-up. Secondly, data collection was restricted to the available information in the patients’ medical records, resulting in some incomplete data for the assessed parameters.

## Conclusion

Coarctation treatment strategy has changed over time: nowadays coarctectomy is performed on pump with aortic arch enlargement with concomitant repair of associated lesions, whereas in older patients with simple coarctation balloon dilatation and stenting has become our preferred approach. Reintervention was required in 40% of patients and was more frequent after catheter-based interventions. Hypertension was present in one out of five patients and more frequent after catheter-based interventions. Our results underscore the importance of regular follow-up with adequate imaging throughout childhood and adulthood after successful repair of aortic coarctation.

### What’s new?

- Patients with more complex aortic coarctation and/or associated cardiac lesions are treated successfully with a more radical approach to concomitant treatment of the aortic-arch.
- Despite improved diagnosis, age at referral has not changed over time.
- Despite evolution of treatment strategies need for reintervention remains frequent.
- Hypertension is common in long-term follow-up despite adequate treatment of coarctation.
